# Effect of thermomechanical ageing on force transmission by orthodontic aligners made of different thermoformed materials: An experimental study

**DOI:** 10.1111/ocr.12825

**Published:** 2024-06-18

**Authors:** Tarek M. Elshazly, Christoph Bourauel, Ahmed M. Ismail, Omar Ghoraba, Philippe Chavanne, Hanaa Elattar, Abdulaziz Alhotan

**Affiliations:** ^1^ Oral Technology, Dental School University Hospital Bonn Bonn Germany; ^2^ Biomaterials Department, Faculty of Dentistry Ain Shams University Cairo Egypt; ^3^ Institut Straumann AG Basel Switzerland; ^4^ Orthodontic Department, Faculty of Dentistry Suez Canal University Ismailia Egypt; ^5^ Department of Dental Health, College of Applied Medical Sciences King Saud University Riyadh Saudi Arabia

**Keywords:** ageing, biomaterials, orthodontic force, removable thermoplastic appliance, tooth movement

## Abstract

**Objectives:**

Investigating the impact of thermal and mechanical loading on the force generation of orthodontic aligners made from various thermoplastic materials and different compositions.

**Materials and Methods:**

Five distinct materials were utilized including, three multi‐layer (Zendura FLX, Zendura VIVA, CA Pro) and two single‐layer (Zendura A and Duran). A total of 50 thermoformed aligners (n = 10) underwent a 48‐hour ageing protocol, which involved mechanical loading resulting from a 0.2 mm facial malalignment of the upper right central incisor (Tooth 11) and thermal ageing through storage in warm distilled water at 37°C. The force exerted on Tooth 11 of a resin model was measured both before and after ageing using pressure‐sensitive films and a biomechanical setup.

**Results:**

Before ageing, pressure‐sensitive films recorded normal contact forces ranging from 83.1 to 149.7 N, while the biomechanical setup measured resultant forces ranging from 0.1 to 0.5 N, with lingual forces exceeding facial forces. Multi‐layer materials exhibited lower force magnitudes compared to single‐layer materials. After ageing, a significant reduction in force was observed, with some materials experiencing up to a 50% decrease. Notably, multi‐layer materials, especially Zendura VIVA, exhibited lower force decay.

**Conclusions:**

The force generated by aligners is influenced by both the aligner material and the direction of movement. Multi‐layer materials exhibit superior performance compared to single‐layer materials, primarily because of their lower initial force, which enhances patient comfort, and their capability to maintain consistent force application even after undergoing ageing.

## 1 INTRODUCTION

Clear aligners present a promising and increasingly popular alternative to traditional orthodontic wire/bracket systems, although ongoing debates persist regarding their effectiveness.^1^ They offer several advantages, including superior aesthetics, improved hygienic and enhanced comfort during treatment.^2^ However, achieving optimal clinical outcomes depends on meticulously optimizing various factors.^3^, ^4^ One critical factor is the selection of an appropriate aligner material, since material behaviour significantly influences aligner performance.^5^ In orthodontic applications, ideal materials should exert continuous light forces over an extended period and return to their original shape after removal from the oral cavity.^4^, ^6^, ^7^ This necessitates a material with sufficient stiffness to apply the required force within the orthodontic physiological limit and a high elastic limit to prevent permanent deformation.^8^

Currently, the most common method for aligner manufacturing involves thermoforming various viscoelastic polymer sheets, including materials like polyethylene terephthalate (PET), polyethylene terephthalate glycol (PET‐G) and thermoplastic polyurethane (TPU).^9^, ^10^ These sheets exhibit viscoelastic properties such as creep and stress relaxation, resulting in significant variations in their mechanical behaviour over time and under loading.^8^, ^11^ Moreover, the mechanical properties of thermoformed sheets differ substantially from their raw sheets counterparts.^12^, ^13^

Patients are instructed to wear aligner splints throughout the day, removing them only during meals and for oral hygiene measures. Typically, each aligner is worn for approximately 2 weeks before being replaced, undergoing intermittent thermal and mechanical loads during this period. Thermal stresses arise from both the typical oral temperature (37°C) and the consumption of hot and cold beverages. Short‐term mechanical loads occur during insertion and removal, while long‐term load results from continuous contact with malaligned teeth.^3^, ^14^

Despite the widespread use of aligners, the existing literature still lacks a comprehensive understanding of aligner biomechanics. To address this gap, our research team is undertaking an extensive project on orthodontic aligners, investigating various aspects at multiple levels. This includes providing insights into how the mechanical properties of aligners, made of different materials, are affected by ageing over different durations, using various ageing agents,^15^ ageing techniques,^16^ and measurement methods.^16^, ^17^ As a part of the project, the aim of the current research is to report the changes in forces generated by different thermoformed aligner materials after undergoing a 2‐days ageing protocol involving both thermal and mechanical loading, with a specific focus on comparing the mechanical behaviour of multi‐layer and single‐layer materials before and after ageing.

## 2 MATERIALS AND METHODS

**FIGURE 1:**
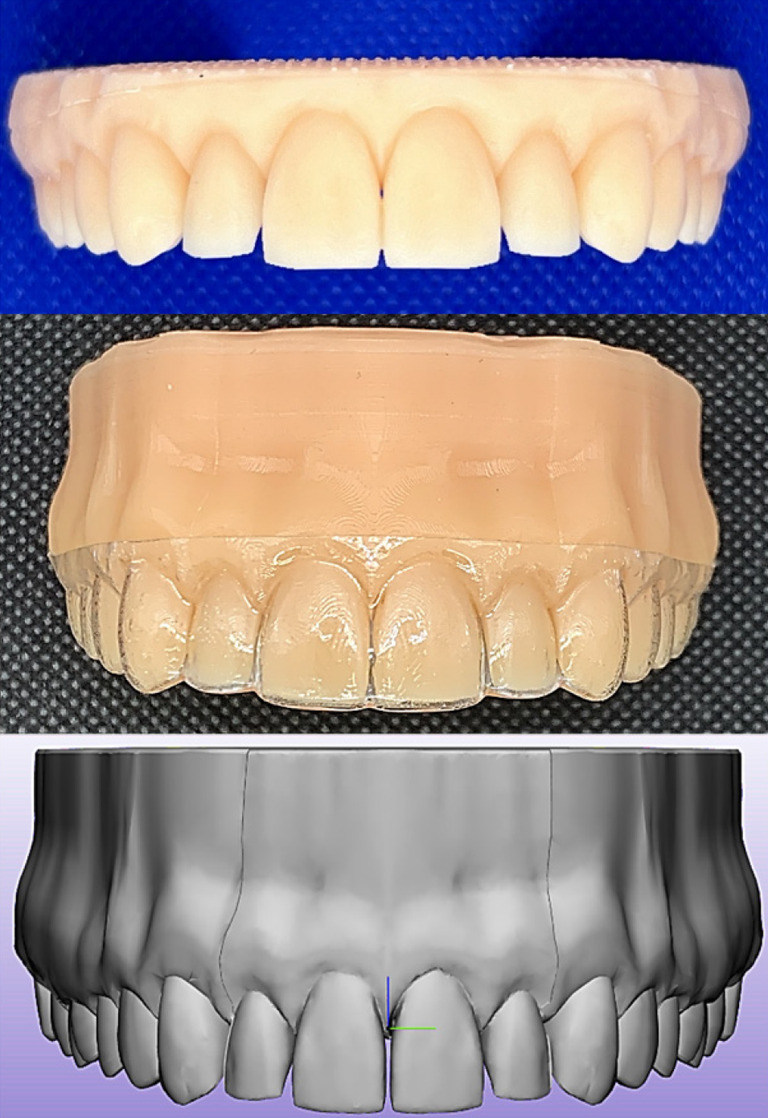
Resin model used for thermoforming with a 15 mm height that has a shallow groove 2 mm beyond the gingival line to facilitate standardization of trimming (upper), an aligner trimmed in straight extended design placed on a printed model (middle), and a digital model with a 100 μm deep housing space for pressure‐sensitive film measurements (lower).

Based on a 3D data set of a full‐dentate maxilla (Digimation Corp., St Rose, Louisiana, USA), a digital model of the upper arch was imported into an image processing software (3‐matic 16.0; Materialise, Leuven, Belgium), where two types of models were designed: one aligned model (for thermoforming purposes) (Figure 1), and a second malaligned model where the upper right central incisor (Tooth 11) was bodily translated facially 0.2 mm (for mechanical ageing purposes). The models were printed using a resin (P pro resin; Straumann, Basel, Switzerland) and a digital light processing (DLP) 3D‐printer (P20+; Straumann, Basel, Switzerland).

Five different types of thermoplastic sheets were investigated (Table 1). Ten aligners (n = 10) were made from each material, following the manufacturers' guidelines, using a thermoforming device (Biostar; Scheu‐dental GmbH, Iserlohn, Germany). All aligners were thermoformed and trimmed (in a straight 2‐mm extended design) by a single trained technician on the same aligned 3D printed model (Figure 1).

**TABLE 1:** Investigated aligner materials; specifying their manufacturer, thermoforming conditions, thickness and composition.

Name	Manufacturer	Thermoforming code/thickness	Thermoforming conditions	Material composition
Zendura FLX®	Bay Materials (Fremont, USA)	Code (162) 0.75 mm	Heating for 50 s at 220°C, and pressure‐forming at 4.8 bar, cooling for 60 s	Three layers sheet of a soft thermoplastic elastomeric layer between two hard layers of co‐polyester
Zendura VIVA®	Bay Materials (Fremont, USA)	Code (162) 0.89 mm	Heating for 50 s at 220°C, and pressure‐forming at 4.8 bar, cooling for 60 s	Three layers sheet of a soft thermoplastic elastomeric layer between two hard layers of co‐polyester
CA® Pro	Scheu‐Dental (Iserlohn, Germany)	Code (112) 0.75 mm	Heating for 25 s at 220°C, and pressure‐forming at 4.8 bar, cooling for 60 s	Three layers sheet of a soft thermoplastic elastomeric layer between two hard layers of co‐polyester
Zendura® A	Bay Materials (Fremont, USA)	Code (162) 0.75 mm	Heating for 50 s at 220°C, and pressure‐forming at 4.8 bar, cooling for 60 s	Single layer sheet of thermoplastic polyurethane (TPU)
Duran®	Scheu‐Dental (Iserlohn, Germany)	Code (112) 0.75 mm	Heating for 25 s at 220°C, and pressure‐forming at 4.8 bar, cooling for 60 s	Single layer sheet of Polyethylene terephthalate glycol‐modified (PETG)

Force transmitted by aligners to Tooth 11 was recorded for each aligner before ageing (Day 0) and after 48 hours of thermomechanical ageing (Day 2). For thermomechanical ageing, the aligner was positioned over the malaligned model and soaked for 48 hours in a temperature‐controlled circulating water bath (DC5‐Haake K15; Thermo‐Haake GmbH, Karlsruhe, Germany) filled with distilled water at 37°C.

**FIGURE 2:**
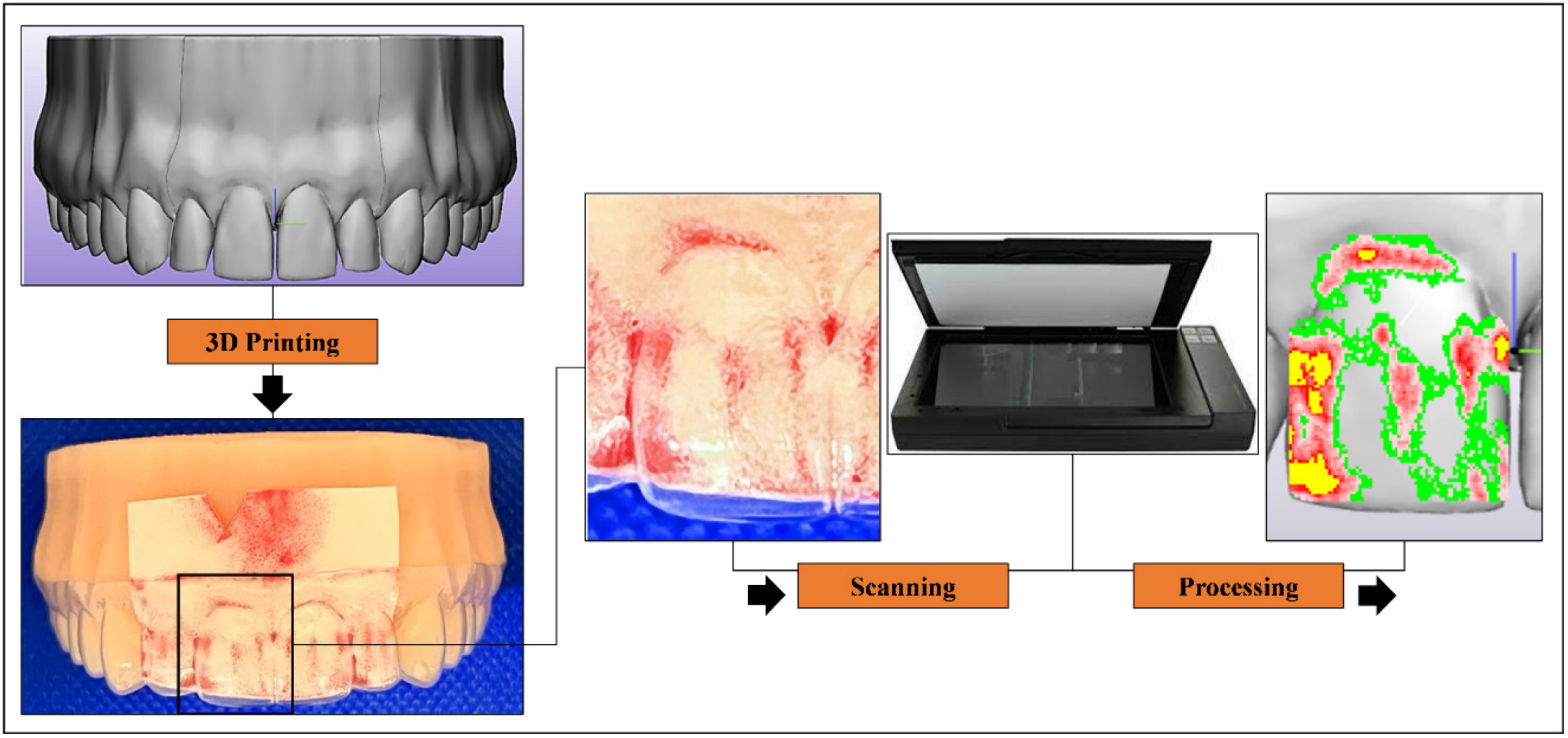
A visual representation of the process for measuring the local contact normal force using Fuji® pressure‐sensitive film on the crown of an upper right central incisor. The pressurized film underwent scanning with an EPSON scanner, and to enhance clarity, the 2D scan was superimposed onto a 3D digital model of the tooth.

**FIGURE 3:**
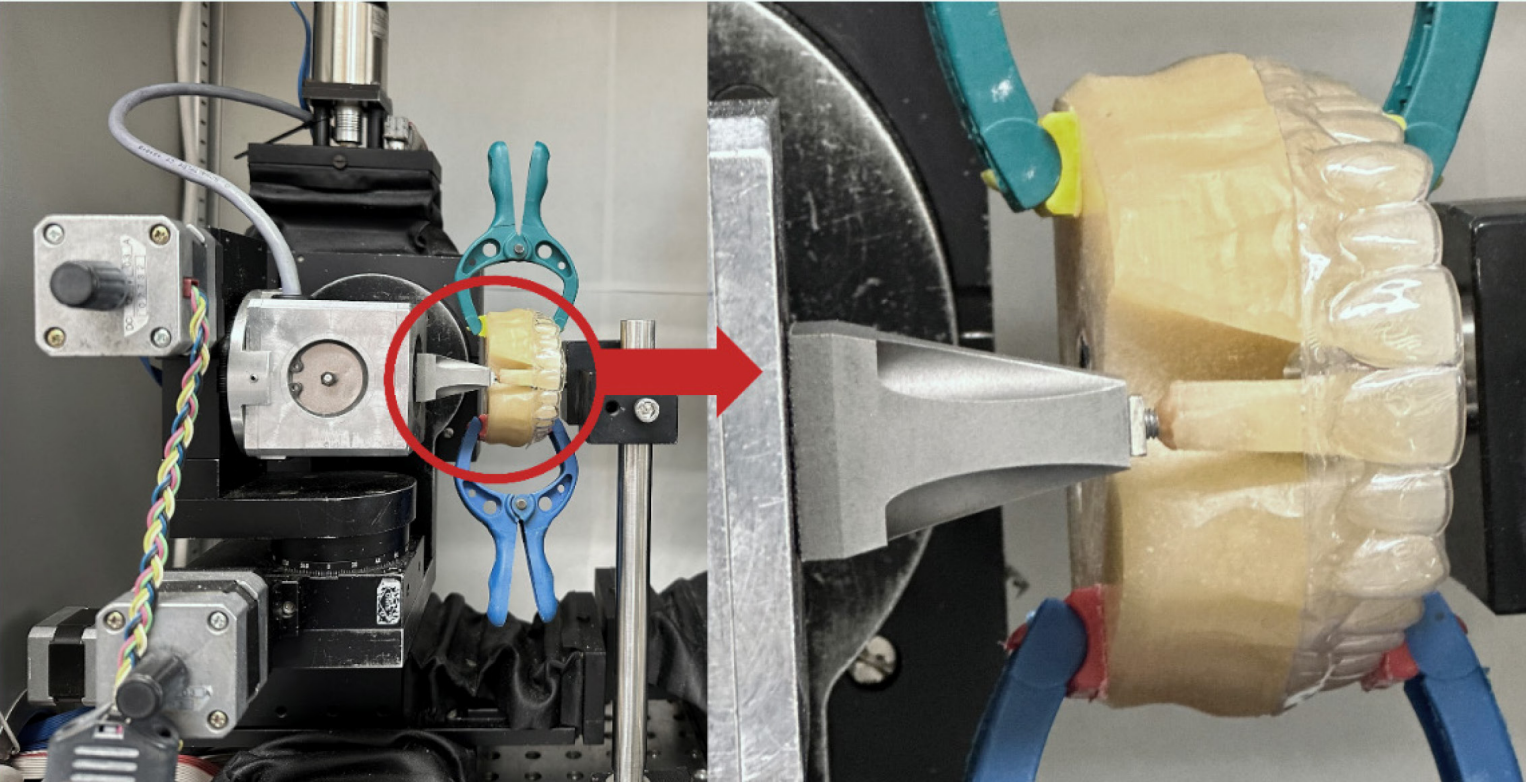
Resin model with an aligner placed into the Orthodontic Measurement and Simulation System (OMSS), where the movable upper right central incisor (Tooth 11) was adjusted to a neutral position in the aligner before measurements.

Force measurements were carried out using two techniques. The first technique involved Low Pressure (LW) pressure‐sensitive films (Fuji® Prescale Film; Fuji Film, Tokyo, Japan) (Figure 2) following the steps reported in a recent study^17^ to record the local contact formal force over the tooth crown. The second technique was an Orthodontic Measurement and Simulation System (OMSS),^18^ a custom‐made biomechanical setup that can replicate 3D tooth movements while measuring the resultant force exerted on the tooth (Figure 3).

For pressure‐sensitive film measurements, an additional malaligned digital model was designed and printed with a created 100 μm space around the four incisors and their corresponding gingival area (housing space) (Figure 1). This housing space was created to exclude the effect of the inherent thickness of the films. The films were carefully seated in the designed housing space, and then the aligner was inserted cautiously to avoid undesirable pressure. Subsequently, each pressurized film underwent scanning using a specialized scanner (EPSON Perfection V300 series; SEIKO Epson CORPORATION, Japan) (Figure 2). Digitalization and quantification of force were performed using coupled software (FPD‐8010E analysis system; Fuji® Film, Tokyo, Japan), which facilitated the direct calculation of force over the tooth surface.

For measurements in the OMSS, an additional duplicate resin model was created, in which Tooth 11 was made movable without any resistance and was attached to the measuring unit, while the resin model was securely fixed (Figure 3). Before each measurement, the tooth was adjusted to its neutral position in the resin model and in relation to the sensor axis, with no forces being applied to the tooth. For measurement, the tooth was translated +0.2 mm facially and −0.2 mm lingually, with a speed of 0.01 mm/second.

## 3 STATISTICAL ANALYSIS

Normality was assessed by examining the data distribution and utilizing the Shapiro‐Wilk test. The data exhibited a parametric distribution and was presented as mean and standard deviation. The analysis was conducted using a one‐way ANOVA followed by a post‐hoc Tukey HSD Test. The significance level (α) was set at *P* ≤ .05. Statistical analysis was carried out using IBM SPSS Statistics Version 25 for Windows (IBM, Endicott, NY, USA).

## 4 RESULTS

**FIGURE 4:**
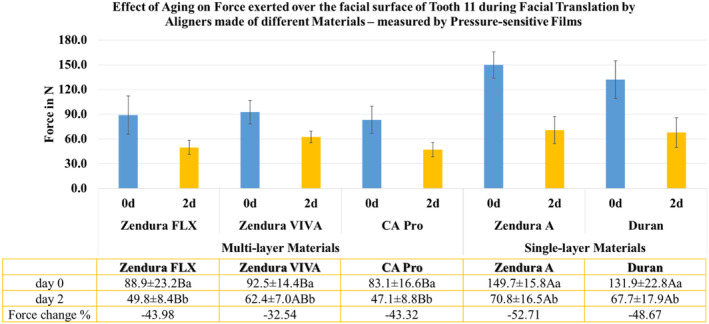
Mean forces of aligners made from different thermoformed sheets exerted on Tooth 11 at two intervals of time (Day 0 and Day 2) recorded using Fuji pressure sensitive films. Different upper and lowercase superscript letters indicate a statistically significant difference within the same horizontal row and vertical column, respectively.

Before ageing (Day 0), the local normal contact force over the entire facial surface of the crown of Tooth 11, as recorded by the pressure‐sensitive films, ranged from 83.1 to 149.7 N. After ageing (Day 2), this range decreased to 47.1 to 70.8 N. The single‐layered materials (Zendura A and Duran) exhibited higher force than the multi‐layered materials (Zendura FLX, Zendura VIVA, and CA Pro). Following 48 hours of thermomechanical ageing, all materials experienced a substantial decrease in force, reaching approximately 50% of force decay, with Zendura VIVA exhibiting the lowest percentage of force decay (32.5%) (Figure 4).

**FIGURE 5:**
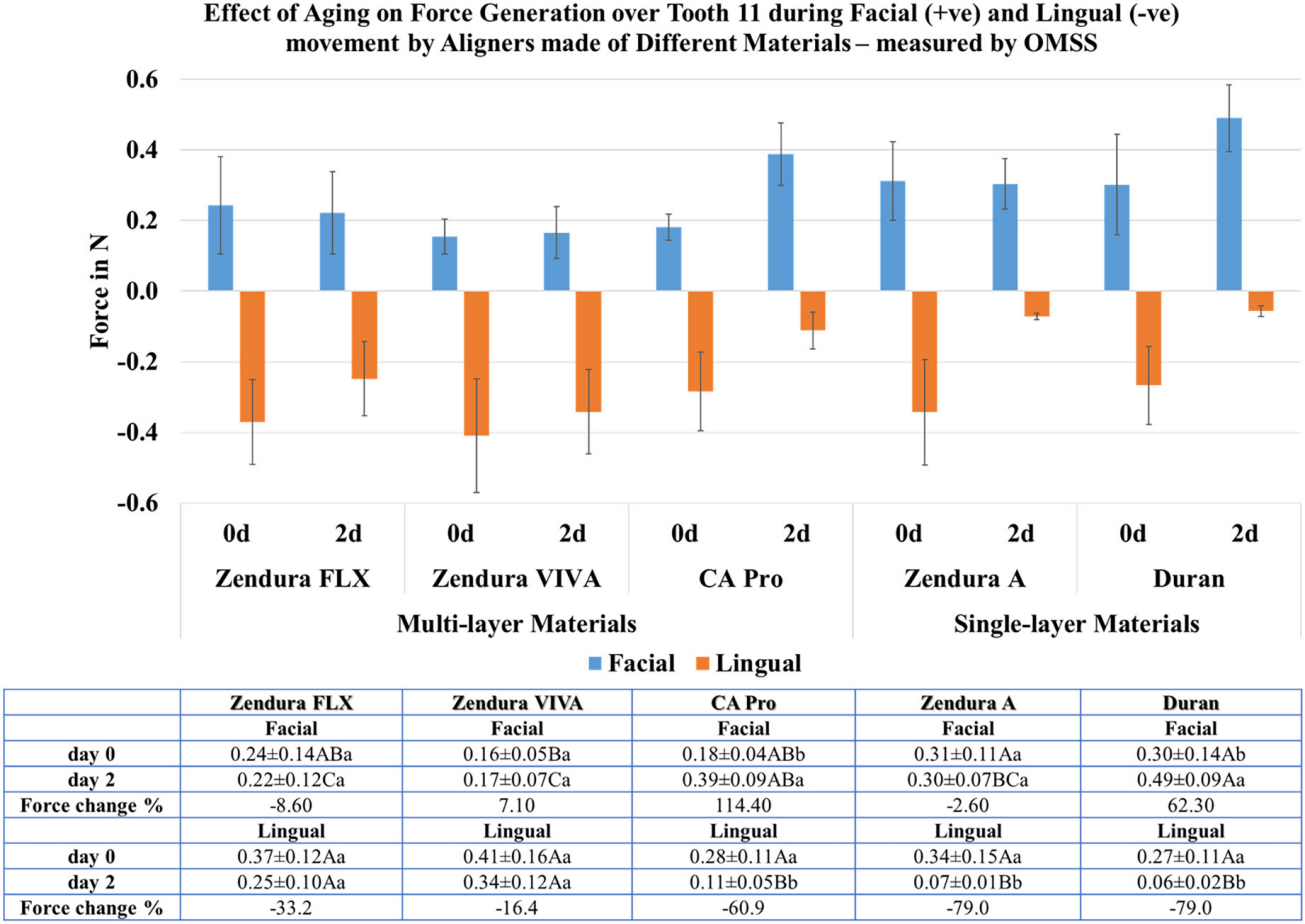
Mean forces of aligners made from different thermoformed sheets exerted on Tooth 11 at two intervals of time (Day 0 and Day 2) recorded using an orthodontic measurement and simulation system (OMSS). Different upper and lowercase superscript letters indicate a statistically significant difference within the same horizontal row and vertical column, respectively.

The OMSS recorded resultant force values ranging from 0.1 to 0.5 N. Force generation exhibited directional dependence, with lingual force surpassing facial force. Before ageing, in the facial direction, single‐layer aligner materials demonstrated significantly higher force values than multi‐layer materials. In contrast, in the lingual direction, no significant differences were observed among all tested materials. Following ageing, in the facial direction, Duran and CA Pro showed a significant increase in force, while the other tested materials maintained their initial force. In the lingual direction, Duran, Zendura A and CA Pro exhibited a significant large force drop of 79.0%, whereas Zendura FLX and Zendura VIVA showed insignificant force decreases of 33.2% and 16.4%, respectively (Figure 5).

## 5 DISCUSSION

Understanding the biomechanical characteristics of orthodontic aligners is crucial for achieving effective and successful treatment outcomes. Additionally, investigating their material properties offers clinicians valuable insights into some of their limitations.^14^ In this study, the impact of thermomechanical ageing on force generation by aligners was investigated, with a particular focus on comparing the mechanical behaviour between multi‐layer and single‐layer materials before and after ageing.

Over a 48‐hours period, the aligners underwent mechanical loading due to the facial malalignment of Tooth 11 and thermal loading by being stored in distilled water at 37°C (oral temperature). Technical constraints provoked using distilled water as an alternative to the artificial saliva for aligner ageing; nonetheless, a prior study reported their comparable effects.^15^ Force measurements were taken both before the ageing process and after 2 days, a chosen ageing period based on previous studies indicating a significant force reduction during the initial 48 hours of aligner use, with no significant difference thereafter.^8^, ^19^, ^20^ Furthermore, both thermal and mechanical loading were employed, as the sole application of thermal ageing has minimal impact on force generation by orthodontic aligners, but the cumulative effects of repeated mechanical loading play a significant role in force decay.^16^, ^21^

Pressure‐sensitive films have gained widespread acceptance in the literature and have been utilized in numerous studies to quantify the contact force between aligners and teeth.^17^, ^22^, ^23^, ^24^ Their technique‐sensitivity and the unavoidable extra forces during aligner insertion and removal were mitigated through careful and meticulous usage, coupled with an expansion in the sample size (n = 10). Barbagallo et al^22^ reported in vivo force values of about 28.0 N, while Cervinara et al^23^ presented pressure values around 3.3 MPa in a comparable in vitro study. Despite the differing units used in the latter study, current force values align with their findings, as the measured pressurized surface area was approximately 40 mm^2^, with force (N) being the product of pressure (MPa) and area (mm^2^).

The force values measured by OMSS for the examined materials fall within the recommended range for orthodontic force, which is 0.7‐1.2 N.^25^ The substantial discrepancy in force values between pressure‐sensitive films and OMSS is attributed to the fundamental principles upon which each method is based. Pressure‐sensitive films provide localized, one‐dimensional force measurements on a single tooth surface, resulting in higher force values, whereas OMSS can measure the 3D forces exerted by the aligner across multiple positions of the crown simultaneously, yielding a lower overall resultant force.

Utilizing biomechanical set‐ups similar to OMSS, different studies^26^, ^27^, ^28^ have reported forces exerted by aligners on Tooth 11. Hahn et al^26^ documented forces of 2.7 N, Elkholy et al^27^ measured forces of 4.5 N, and Kohda et al^28^ reported forces of 2.9 N. Using OMSS, Simon et al^29^ recorded force levels ranging from 0.2 to 1.5 N, consistent with the present study's findings. Discrepancies in values between OMSS and comparable devices may arise from the use of an ideal centre of resistance for the measured tooth in OMSS. Furthermore, differences in the measurement protocol, material composition,^5^ trimming line design,^17^ extent and type of tooth activation, and aligner thickness^30^ could also contribute to variations across studies.

Consistent with Lombardo et al,^8^ the current study showed higher forces in single‐layered sheets, reflecting increased rigidity, while multi‐layered sheets exhibited lower forces, indicative of higher flexibility. Moreover, a considerable reduction in force with aligners is noted following 48 hours of ageing. Additionally, single‐layer materials experienced significantly higher force decay compared to multi‐layer counterparts. When aligner sheets are immersed in water, the absorption of water molecules into the plastic through diffusion between polymer chains, encouraged by elevated temperatures, increases polymer mobility, and performs a plasticizing effect which eventually leads to weakening of the plastic.^8^, ^31^, ^32^

In the present study, the simultaneous application of thermal and mechanical loads resulted in a force decrease, consistent with prior findings.^16^, ^21^ However, an increase in force generation in the facial direction was observed in Duran and CA Pro with OMSS. This can be attributed to the cold working of facial segments corresponding to the malaligned tooth's location, leading to increased hardness.^33^ In simpler terms, PET‐G based sheets undergo strain‐induced crystallization under deformation.^34^ In the lingual direction, Zendura FLX and Zendura VIVA demonstrated sustained force generation post‐ageing, with Zendura VIVA exhibiting the lowest recorded percentage of force decay. This observation may be attributed to a unique composition of Zendura VIVA, which displays resilience against the ageing process.

This experimental study has some limitations, including the absence of representation for the periodontal ligament and bone, which play a significant role in the stress distribution of orthodontic appliances. Nevertheless, this factor can be adequately addressed, given that we are evaluating the performance of different materials under identical conditions. Future studies could explore various tooth movements of Tooth 11 and different teeth. Furthermore, the current method could facilitate a comprehensive analysis of 3D printed aligners compared to traditional thermoformed aligners.

## 6 CONCLUSION

The current in vitro study yielded the following observations:

1. Force generation during aligner splint deformation over a malaligned tooth is significantly influenced by the type of aligner material and the direction of movement.
2. Three‐layer aligner sheets such as Zendura FLX, Zendura VIVA and CA Pro exhibit lower initial forces on teeth compared to the single‐layer sheets like Zendura A and Duran.
3. Thermomechanical ageing of aligners results in a significant force decay within the first 48 hours.
4. The impact of ageing is less pronounced in multilayer materials than in single‐layer ones.
5. Mechanical loading from tooth malalignment may induce cold working and material hardening in some aligner materials, leading to an increase in force generation.

## AUTHORS CONTRIBUTIONS

Tarek M. Elshazly: Writing – review and editing, Writing – original draft, Visualization, Validation, Resources, Data curation. Christoph Bourauel: Writing – review and editing, Visualization, Supervision, Resources, Project administration, Data curation, Conceptualization. Ahmed Ismail: Writing – original draft, Methodology, Investigation, Formal analysis. Omar Ghoraba: Methodology, Investigation. Philippe Chavanne: Writing – review and editing, Visualization. Hanaa Elattar: Writing – review and editing, Visualization. Abdulaziz Alhotan: Writing – review and editing, Formal analysis, Data curation. All authors have read and agreed to the published version of the manuscript.

## FUNDING INFORMATION

Part of this work was supported by Straumann AG, Basel, Switzerland.

## CONFLICT OF INTEREST STATEMENT

The authors declare that they have no known competing financial interests or personal relationships that could have appeared to influence the work reported in this paper.

## COMPLIANCE WITH ETHICS REQUIREMENTS

This article does not contain any studies with human or animal subjects.

## INSTITUTIONAL REVIEW BOARD STATEMENT

Not Applicable.

## INFORMED CONSENT STATEMENT

Not Applicable.

## USE OF LARGE LANGUAGE MODELS

ChatGPT was used to improve the English language of the current paper and to rephrase some parts to a more understandable way.

## Data Availability

Data will be made available on request.
